# Effect of *CYP3A4* Methylation on Tacrolimus Pharmacokinetics

**DOI:** 10.1097/FTD.0000000000001351

**Published:** 2025-07-08

**Authors:** Karel K. M. Koudijs, Oumaima Etsouli, Costanza L. Vallerga, Dirk Jan A. R. Moes, Bastian N. Ruijter, Jesse J. Swen, Minneke J. Coenraad, Teun van Gelder

**Affiliations:** * Department of Clinical Pharmacy and Toxicology, Leiden University Medical Center, Leiden, the Netherlands;; † Department of Internal Medicine, Erasmus Medical Center, Rotterdam, the Netherlands;; ‡ Leiden Transplant Center, Leiden University Medical Center, Leiden, the Netherlands; and; § Department of Gastroenterology, Hepatology and Transplantation Center, Leiden University Medical Center, Leiden, the Netherlands.

**Keywords:** tacrolimus, transplantation, liver, pharmacokinetics, therapeutic drug monitoring

## Abstract

**Background:**

Common genetic variants in *CYP3A4* together only explain a limited amount of the variability in tacrolimus clearance. This cross-sectional study aimed to explore the extent to which pharmacokinetic variability can be explained by methylation of the *CYP3A4* gene.

**Methods:**

Residual tissue material from liver biopsies routinely collected 6 months post-transplantation was used. Inclusion criteria were tacrolimus once daily (Advagraf) in a steady state (ie, no dose change in the previous 3 days); assessment of tacrolimus pharmacokinetics within 3 weeks of the biopsy; and no documented episode of rejection for at least 3 months prior. Patients and liver donor tissue were genotyped. Only patients in which the patient and the donor had a genotype that did not express the CYP3A5 protein were included. The liver biopsy tissue material was then analyzed using an Illumina Infinium MethylationEPIC array.

**Results:**

Of the 28 patients who met the inclusion criteria, 23 passed the quality control assessment required for the methylation analysis. Increased methylation of 1 of the 10 methylation probes within the *CYP3A4* gene region (cg19046783) was positively correlated (Spearman correlation coefficient, 0.52) with the dose-normalized area under the concentration versus time curve (AUC)_0–24h_ (*P* = 0.01). When quantified using univariate linear regression, this probe explained 18% of the variation in the dose-normalized AUC_0–24h_. Interestingly, cg19046783 had the lowest mean methylation and highest biological variation.

**Conclusions:**

Increased methylation of 1 of the 10 methylation probes within the *CYP3A4* gene region (cg19046783) was positively correlated with increased dose-normalized AUC_0–24h_, which explained 18% of the variation in the dose-normalized AUC_0–24h_ using univariate linear regression.

## Abstract 2

Supplemental Digital Content is Available in the Text.

## INTRODUCTION

Tacrolimus is the most frequently prescribed calcineurin inhibitor for solid-organ transplantation. It is considered to be a narrow therapeutic index drug, and therapeutic drug monitoring is routinely performed to prevent underexposure and overexposure. The substantial between-patient variability in the pharmacokinetics of tacrolimus is partly explained by patient age, donor and recipient ethnicity, hematocrit, corticosteroid dosage, gut bacteria and cytochrome P450 isoenzyme, and P-glycoprotein genetics.^1–3^ Polymorphisms in the *CYP3A*5 gene have been reported to be particularly clinically relevant for the dose requirement of tacrolimus.^4^ The Clinical Pharmacogenetics Implementation Consortium recommends increasing the starting dose of tacrolimus in patients with an extensive or intermediate CYP3A5 metabolizer phenotype to 150%–200% of the regular dose.^5^ In addition to *CYP3A5* gene polymorphisms, it has been reported that additional interindividual variability in tacrolimus clearance can be explained by common single nucleotide variants in *CYP3A4*, such as CYP3A4*22.^6–8^ Furthermore, since CYP3A4 and CYP3A5 proteins are both expressed in the liver and intestine, donor and recipient genetics are important for liver transplantation.^9^

Despite numerous population-level pharmacokinetic studies and extensive pharmacogenetic analyses, only 50% of the between-patient variability in tacrolimus pharmacokinetics can be explained by genetics.^10,11^ Food–drug interactions or the influence of the gut microbiome on drug clearance may play a role.^12^ Another factor that may be responsible for the unexplained between-patient variability is epigenetic change. In epigenetics, the base sequences in gene DNA are unaltered; however, nucleotides may be methylated.^13^ DNA methylation may affect transcriptional regulation through several processes, including transcriptional suppression or histone modification. The regulation of DNA methylation is mainly attributed to CpG islands, which are composed of clusters of CpG sites (ie, regions in the DNA sequence where cytosine is followed by guanine in a specific direction). As a result of these epigenetic changes, protein expression may change, and for metabolizing enzymes, the clearance of a drug may increase or decrease.^14^

DNA methylation is tissue specific. Therefore, to study the impact of epigenetics on tacrolimus pharmacokinetics, it is crucial to study changes in the DNA of hepatocytes. Relevant tissue-specific materials are not readily available because collecting liver biopsies for pharmacokinetic studies is considered to be ethically challenging. However, at our center, at 6 months after liver transplantation, patients undergo a protocol liver biopsy. Furthermore, a few days prebiopsy or postbiopsy, the exposure to tacrolimus is monitored by measuring the area under the concentration versus time curve (AUC).

To the best of our knowledge, the effects of *CYP3A4* methylation on clinical tacrolimus exposure have not been investigated. Therefore, we performed an exploratory study to investigate the degree to which *CYP3A4* methylation, as measured in the protocol liver biopsy 6 months post-transplantation, can explain the between-patient variability in the tacrolimus AUC.

## MATERIALS AND METHODS

### Study Cohort

The study protocol and use of patient material was approved by the Medical Ethical Review Committee “Leiden—Den Haag—Delft” (METC LDD) under protocol ID: “B21.004.” All patients provided oral and written informed consent for participation in the Gastroenterology and Hepatology Biobank, and for the use of their personal data and liver biopsy tissue samples for scientific research. Liver donor-to-recipient matching was performed using Eurotransplant allocation rules. The Strengthening of Reporting of Observational Studies in Epidemiology cross-sectional reporting guidelines checklist was used to check and construct this report.^15^

The inclusion criteria were adult liver transplantation, with once-daily tacrolimus (Advagraf; Astellas Pharma, Tokyo, Japan) maintenance therapy, assessment of tacrolimus pharmacokinetics within 3-week window from the liver biopsy, in a steady state (ie, at least 3 days after the last dose change), and without documented episodes of rejection within 3 months before the biopsy.

### Genotyping

The DNA of liver transplantation donors (all deceased donors) and eligible liver recipients was genotyped for CYP3A4*22 (rs35599367), CYP3A5*3 (rs776746), and CYP3A5*6 (rs10264272). CYP3A5*7 was not typed because it is not present in individuals of European ancestry.^16^ To exclude the effect of CYP3A5 on tacrolimus clearance, only patients in which the liver donor and the recipient had a genotype that did not express the CYP3A5 protein (ie, CYP3A5*3/*3) were included.

### Therapeutic Drug Monitoring

Blood tacrolimus levels were determined using a previously validated liquid chromatography-tandem mass spectrometry assay that is capable of simultaneously determining tacrolimus, sirolimus, everolimus, and cyclosporine.^9^ All the parameters were in accordance with the bioanalytical method validation guidelines of the European Medicines Agency (Committee for Medicinal Products for Human Use, CHMP 2011, Guidelines on bioanalytical method validation). AUC_0–24h_ maximum a posteriori Bayesian estimation was performed using MW/Pharm version 3.83 (Mediware, Groningen, the Netherlands), based on a population pharmacokinetic model for once-daily tacrolimus, C_0h_, C_2h_, C_3h_, yielding the estimated AUC from time zero to 24 hours (AUC_0–24h_).^9^

### Generation of Methylation Array Data

To assess the quality of the molecular DNA, 5% of the samples were randomly selected and assessed using agarose gel imaging. Subsequently, the DNA samples were transported to the Human Genomics Core Facility of the Department of Internal Medicine at the Erasmus Medical Center. DNA samples were placed in 96-well PCR plates with 15 µL of DNA at a concentration of 50 ng/µL.

DNA methylation was measured using the genome-wide methylation array Infinium MethylationEPIC BeadChip (Illumina, San Diego, CA) with >850k CpG sites, according to the manufacturer's protocol. Briefly, 500 ng of genomic DNA was converted with bisulfite using the EZ-96 DNA Methylation MagPrep Kit (Zymo Research, Irvine, CA) with a KingFisher Flex robot (Thermo Fisher Scientific, Breda, the Netherlands). The samples were then plated in a randomized order. Bisulfite conversion was performed according to the manufacturer's protocol, with the modification of 15 µL MagBinding Beads to bind the DNA. The conversion reagent incubation was performed according to the following cycle protocol: 16 cycles of 95°C for 30 seconds followed by 50°C for 1 hour. After the cycle protocol, the DNA was incubated for 10 minutes at 4°C. Next, DNA samples were hybridized on the Infinium MethylationEPIC v1.0 BeadChip (Illumina), according to the manufacturer's protocol with a modification of 8 µL bisulfite-treated DNA as the start material.

### Data Preprocessing and Statistical Analysis

All preprocessing and statistical analyses were performed using R (version 4.2.2; R Foundation for Statistical Computing, Vienna, Austria). Two-sided *P* < 0.05 was considered to be statistically significant. Because of the exploratory nature of the study and the limited sample size, *P*-values were not corrected for multiple testing. All the code and anonymized data required to reproduce these analyses are available from the following GitHub repository: https://github.com/kkmkoudijs/Effect_of_CYP3A4_methylation_on_tacrolimus_pharmacokinetics.

The methylation data were imported and preprocessed using the methylationArrayAnalysis R package (version 1.22.0), conforming to the standard workflow as presented in the accompanying vignette (Bioconductor, 2024, methylationArrayAnalysis R package vignette). The example workflow was altered to accommodate the use of a different methylation platform. In summary, the “IlluminaHumanMethylationEPICanno.ilm10b5.hg38,” “IlluminaHumanMethylationEPICmanifest,” and minfi R packages were used to convert the raw data (IDATs) into an “RGChannelSet” object that was amenable to further processing in R. First, only samples with mean detection of *P* < 0.05 for all probes were included. Second, a QC report was generated and inspected using the qcReport function. After inspection of the QC report, the data were quantile-normalized using the preprocessQuantile function (see **Figure**, **Supplemental Digital Content 1**, http://links.lww.com/TDM/A862). Next, any probe that failed in 1 or more samples was removed using a detection of *P* > 0.01; this step removed 36,391 of 865,859 probes (4.2%). Finally, the analyses were restricted to the 10 CpG probes included in the *CYP3A4* gene region.

As a proxy for the tacrolimus-metabolizing activity of each patient, the dose-normalized area under the tacrolimus AUC over the previous 24 hours (AUC_0–24h,norm_) was used, which was calculated using the following formula:

$${\text{AUC}}_{0-24h,\text{norm}}=\frac{{\text{AUC}}_{0-24h}}{\text{Tacrolimus}\;\text{dose}\;\text{in}\;µg}$$

A previous analysis of 1573 dose-normalized tacrolimus trough concentrations in liver transplantation patients identified 3 statistically significant covariates: hematocrit, the logarithm of the alanine aminotransferase (ALAT) level, and a C-reactive protein (CRP) level >49 mg/L.^17^ In addition, patients with the CYP3A4*22 T-variant allele required a lower dose to achieve the same exposure.^6^ Therefore, these 3 clinical covariates and 2 genotype variables (recipient and donor CYP3A4*22) were tested as potential confounders in the relationship between probe methylation and AUC_0–24h,norm_.

Finally, nonparametric Spearman regression was used as the primary method to test for an association between probe methylation and AUC_0–24h,norm_. The advantage of Spearman regression is that it does not assume a linear relationship; instead, it tests whether there is a monotonically increasing or decreasing relationship between the variables.

## RESULTS

Of the 42 biobank samples that met the pregenotyping inclusion criteria between 2010 and 2020, 14 were excluded because genotyping indicated that the liver recipient or the liver sample had active CYP3A5. Of the remaining 28 liver samples, 23 passed the quality control assessment and 5 failed because of insufficient DNA. The characteristics of the included liver recipients and their associated liver donors are shown in Table 1. It should be noted that, although the mean CRP level is relatively high, 17 of the 23 patients had a normal (ie, <5 mg/L) CRP level. In addition, 4 patients had signs of unstable liver function (total bilirubin, aspartate aminotransferase, or ALAT greater than 2 times the upper limit of normal).

**TABLE 1.** Characteristics of Liver Recipients and Associated Donors for Patients With Methylation Data

	Characteristic	Value (± SD or n)
Liver recipient	Age	52 ± 12 yrs
	Sex	Female (n = 7), male (n = 16)
	Weight	81 ± 18 kg
	Height	180 ± 10 cm
	AUC_0–24h_	240 ± 71 µg*24 h/L
	Dose/d	5.5 ± 2.4 mg
	eGFR	32 ± 26 mL/min
	Hematocrit	0.38 ± 0.046 L/L
	Hemoglobin	7.5 ± 1.1 mmol/L
	Albumin	45 ± 2.7 g/L
	ALAT	47 ± 49 U/L
	ASAT	32 ± 26 U/L
	GGT	82 ± 79 U/L
	Total bilirubin	12 ± 8.4 µmol/L
	Alkaline phosphatase	130 ± 63 U/L
	CRP	5.8 ± 12 mg/L
	Prednisone/prednisolone use	No (n = 17), yes = (n = 6)
	Azole use (eg, ketoconazole)	No (n = 23)
	Transplant dates	2013–2019
	Time between transplant and AUC	180 ± 7.5 d
	Time between AUC and biopsy	−0.22 ± 1.2 d
Liver donor	Sex	Female (n = 11), male (n = 12)
	CYP3A4*22 genotype	C/C (n = 19), C/T (n = 4)

ASAT, aspartate aminotransferase; eGFR, estimated glomerular filtration rate; GGT, gamma-glutamyl transferase.

To determine whether the association between probe methylation and AUC_0–24h,norm_ could be confounded by the known covariates, CYP3A4*22 C/T genotype, hematocrit level, high CRP level (>49 mg/L), and log(ALAT) level, these variables were tested as predictors of AUC_0–24h,norm_ using a linear regression model (see **Table**, **Supplemental Digital Content 1**, http://links.lww.com/TDM/A862). However, none of the tested variables had a statistically significant association with AUC_0–24h,norm_ and were therefore excluded from further analysis.

All 10 methylation probes measuring the *CYP3A4* gene (see **Table**, **Supplemental Digital Content 1**, http://links.lww.com/TDM/A862) passed the quality control assessment. As the beta values of the correlation coefficients among probes were relatively weak (between −0.3 and 0.66 with a median of 0.24 (see **Table, Supplemental Digital Content 1**, http://links.lww.com/TDM/A862), the probes were not combined and the association between the beta values of each of the 10 probes and the AUC_0–24h,norm_ was assessed separately.

**FIGURE 1.**
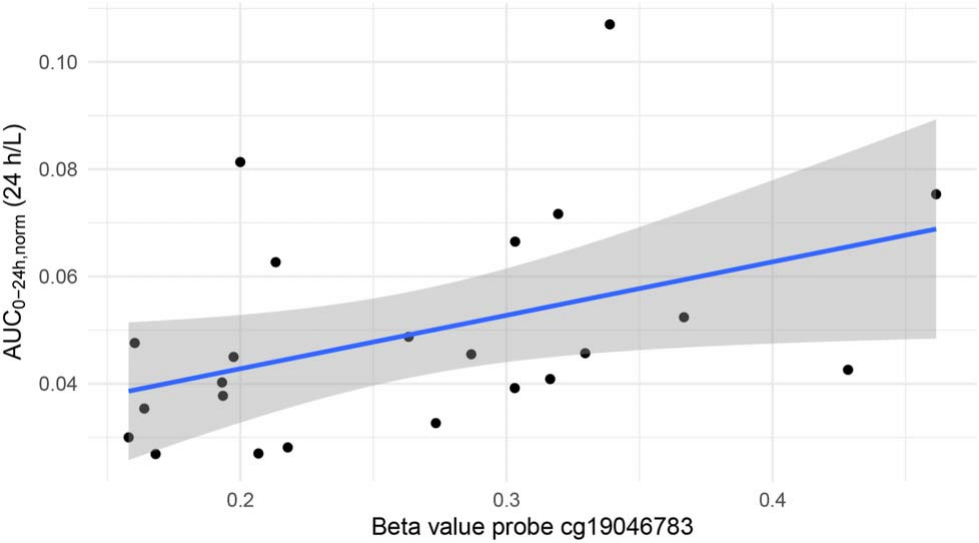
Area under the concentration versus time curve (AUC)_0–24h,norm_ plotted against the beta values of the probe. Note: The solid blue line represents the linear model fit, the shaded gray area represents the 95% confidence interval of the fit. This fit represents a Pearson (ie, linear) correlation coefficient of 0.43 (*P* = 0.04, R^2^ = 18% with 95% confidence interval between 0.04% and 51%). Note: AUC_0–24h,norm_ = dose-normalized AUC_0–24h._

For interpretation of the results, it is important to note that the beta value of a probe can range from 0 to 1, with 1 corresponding to full methylation. Of the 10 probes, cg19046783 was the only probe that had a statistically significant correlation with the AUC_0–24h,norm_ (Table 2). This probe could be further distinguished from the other probes because of its comparatively lower mean beta value (0.26) and higher SD (0.086). The relationship between cg19046783 probe methylation and AUC_0–24h,norm_ is relatively linear (Fig. 1). The AUC_0–24h,norm_ plotted against the beta values of the other 9 probes is shown in **Figure, Supplemental Digital Content 1** (http://links.lww.com/TDM/A862).

**TABLE 2.** Results of Regression of AUC_0–24h,norm_ on the Methylation Levels of the Included Probes

Probe ID	Mean Beta Value ±SD	Spearman Rho (*P*)
cg01526453	0.45 ± 0.09	+0.27 (*P* = 0.20)
cg04358264	0.68 ± 0.04	+0.12 (*P* = 0.57)
cg08653918	0.77 ± 0.04	+0.39 (*P* = 0.07)
cg09914773	0.70 ± 0.05	−0.08 (*P* = 0.73)
cg14770351	0.59 ± 0.04	−0.24 (*P* = 0.26)
cg19046783	0.26 ± 0.09	+0.52 (*P* = 0.01)*
cg20572918	0.89 ± 0.02	−0.32 (*P* = 0.14)
cg22821554	0.86 ± 0.02	−0.15 (*P* = 0.48)
cg23326197	0.66 ± 0.05	−0.14 (*P* = 0.53)
cg24014584	0.63 ± 0.05	+0.10 (*P* = 0.64)

Note: AUC_0–24h,norm_ = dose-normalized AUC_0–24h._

* *P* < 0.05.

## DISCUSSION

Although the *CYP3A5* genotype of the donor and the recipient and age are predictors of the clearance of tacrolimus in recipients of a liver transplantation, a large proportion of the variability in the pharmacokinetics of tacrolimus cannot be explained. We hypothesized that methylation of *CYP3A4* in the liver may be responsible for at least a component of this unexplained variability.

This study shows that 1 of 10 probes in the methylation array on the *CYP3A4* gene had a significant correlation with the tacrolimus exposure data. The positive Spearman correlation coefficient implies that the tacrolimus AUC_0–24h,norm_ increases as methylation of the cg19046783 probe increases, which is the expected direction, as higher methylation implies less gene expression and less CYP3A4 enzyme. With less CYP3A4 enzyme available to metabolize tacrolimus, tacrolimus exposure will increase. The weak correlations observed for the other 9 probes are not unexpected, as the cg19046783 probe has a much lower mean beta value (0.26 for the cg19046783 probe versus a mean of 0.69 for the other probes) and higher SD (0.09 for the cg19046783 probe versus a mean of 0.05 for the other probes). In general, lower standard deviations imply a lower likelihood of explaining a significant amount of between-patient variability in gene expression because this probe location seems to be more biologically stable. Our results are unique because they correlate the methylation status of *CYP3A4* in the liver with a functional CYP3A4 activity test (reflected in tacrolimus pharmacokinetics).

The epigenetic induction and downregulation of *CYP3A4* may result from a variety of physiological and pathophysiological conditions. Methylation of the 5′-position of cytosine residues is a reversible covalent modification of DNA associated with gene repression.^18^ Changes in the methylation of cytosines in the proximal promoters of *CYP3A4* have been linked to developmental changes in the expression of *CYP3A7* in fetal and neonatal livers to *CYP3A4* in the first months after birth.^19^ In the human hepatoma cell line, HepG2, low expression of *CYP3A4* was induced by exposing the cells to the DNA methylation inhibitor, 5-aza-20-deoxycytidine.^20^ In human liver samples, variable CpG methylation sites correspond to *CYP3A4* transcription factor binding sites.^21^ Environmental factors, such as alcohol consumption, inflammation, or drug treatment, may influence the degree of methylation of DNA-encoding liver enzymes, including metabolizing proteins such as CYP3A4. Inflammation has been shown to affect *CYP3A4* gene activity, for example, in critically ill children in whom the CRP level was related to clearance of midazolam, a drug used for quantifying CYP3A‐mediated clearance.^22^ A recent study using Mendelian randomization analysis suggested that altered CpG methylation was a consequence of an increased blood CRP level.^23^

To achieve adequate immunosuppression in the immediate post-transplantation period, it is important that the tacrolimus level should reach the desired target concentration as soon as possible. When a standard tacrolimus starting dose is used (often 0.1–0.2 mg/kg), patients who express CYP3A5 (extensive and intermediate metabolizers) generally have a lower tacrolimus predose concentration compared with patients who do not express CYP3A5 (poor metabolizers). Thus, taking the *CYP3A5* genotype into account to reduce the number of required dose adaptations could be cost-effective and patient-friendly. The Clinical Pharmacogenetics Implementation Consortium issued guidelines for dosing based on the *CYP3A5* genotype.^5^ It is not expected that the epigenetic data obtained from liver biopsies will also be used to determine tacrolimus doses in future patients. A liver biopsy just before transplantation is a highly invasive procedure, not without risk, and the turnaround time for epigenetic analysis is too lengthy to inform the starting dose of tacrolimus. Furthermore, therapeutic drug monitoring is performed intensively after the initiation of tacrolimus treatment, and the dose can be rapidly adjusted based on the measured concentration.

It is most likely that the methylation status of the *CYP3A4* gene is not a fixed patient characteristic but is subject to change over time. Recipients of organ transplantations are exposed to different levels of renal or liver dysfunction (eg, moving from an internal milieu of uremia during dialysis to good renal function after successful kidney transplantation) and episodes of inflammation (infectious complications and rejection of the transplanted organ), which may affect the methylation status of DNA. Repetitive liver biopsies performed over a prolonged follow-up period would allow the study of these changes; however, the invasiveness of this procedure and associated risks are considered to be unethical.

To the best of our knowledge, this is the first study to correlate *CYP3A4* methylation with the clinical measurements of tacrolimus pharmacokinetics. As such, it comes with a few limitations. First, because this to deemed be an exploratory study, a formal sample size calculation or correction for multiple testing were not performed. Second, 4 of the 23 patients could be categorized as having unstable liver function (total bilirubin, aspartate aminotransferase, or ALAT greater than twice the normal limit), and 6 patients had signs of liver inflammation (a CRP level >5 mg/L). Because of the limited sample size, these patients were not excluded. Instead, inspired by a previous analysis, the variables log(ALAT) and CRP >49 mg/L were assessed for an association with AUC_0–24h,norm_.^17^ As these variables were not statistically significantly associated in this limited sample dataset, they were unlikely to act as strong confounders. It should be further emphasized that only 1 patient in our dataset had a CRP level >49 mg/L, with the next highest being 10 mg/L; therefore, inflammation is unlikely to have a significant effect in this cohort. Third, we did not collect or analyze data on donor and recipient ethnicities, concomitant food administration, or alcohol consumption. Therefore, these factors cannot be formally excluded as potential confounders. However, the potential confounding effects of ethnicity were minimized by including only patients who did not express CYP3A5. Fourth, because this study was conducted in a Dutch liver transplantation cohort of mostly Caucasian participants, our findings may not be representative of other ethnicities. Finally, intestinal metabolism may contribute up to 50% of the total metabolism of oral tacrolimus,^24^ potentially reducing the explanatory power of the measured methylation level in the liver. We expect that the methylation level in the liver and intestine are correlated because of their shared environmental influence; however, it is not possible to quantify the correlation between these factors.

A more extensive study is required to replicate these findings and to more reliably exclude the potential impact of confounders. However, this requires the availability of adequately stored liver biopsies, collected concurrently with reliable assessments of tacrolimus pharmacokinetics, preferably in the form of an AUC. To simplify the design of future studies and enhance reproducibility, we have included the statistical code and anonymized data (ie, probe methylation data and dose-normalized AUCs) in a GitHub repository. Furthermore, although these findings may not directly influence the clinical management of tacrolimus dosing protocols, they contribute to the fundamental knowledge on between-patient variability in the pharmacokinetics of tacrolimus and provide avenues for future studies.

## CONCLUSION

In conclusion, we have demonstrated that the methylation status of the *CYP3A4* gene may be associated with between-patient differences in the pharmacokinetics of tacrolimus. In this study, the methylation changes detected using the cg19046783 probe had the strongest correlation with the AUC of tacrolimus. These epigenetic changes could explain a component of the unexplained variability in tacrolimus pharmacokinetics. However, larger studies are required to validate and more precisely quantify what component of the variability is explained by the differential methylation patterns of the *CYP3A4* gene.

## Supplementary Material

## References

[1] Staatz CE Tett SE. Clinical pharmacokinetics and pharmacodynamics of tacrolimus in solid organ transplantation. Clin Pharmacokinet. 2004;43:623–653. 15244495 10.2165/00003088-200443100-00001

[2] Brunet M van Gelder T Åsberg A Therapeutic drug monitoring of tacrolimus-personalized therapy: second consensus report. Ther Drug Monit. 2019;41:261–307. 31045868 10.1097/FTD.0000000000000640

[3] Guo Y Crnkovic CM Won KJ Commensal gut bacteria convert the immunosuppressant tacrolimus to less potent metabolites. Drug Metab Dispos. 2019;47:194–202. 30598508 10.1124/dmd.118.084772 PMC6367689

[4] Nuchjumroon A Vadcharavivad S Singhan W Comparison of tacrolimus intra-patient variability during 6-12 Months after kidney transplantation between CYP3A5 expressers and nonexpressers. J Clin Med. 2022;11:6320. 36362548 10.3390/jcm11216320 PMC9658797

[5] Birdwell KA Decker B Barbarino JM Clinical pharmacogenetics implementation Consortium (CPIC) guidelines for CYP3A5 genotype and tacrolimus dosing. Clin Pharmacol Ther. 2015;98:19–24. 25801146 10.1002/cpt.113 PMC4481158

[6] Hesselink DA Bouamar R Elens L The role of pharmacogenetics in the disposition of and response to tacrolimus in solid organ transplantation. Clin Pharmacokinet. 2014;53:123–139. 24249597 10.1007/s40262-013-0120-3

[7] Andreu F Colom H Elens L A new CYP3A5*3 and CYP3A4*22 cluster influencing tacrolimus target concentrations: a population approach. Clin Pharmacokinet. 2017;56:963–975. 28050888 10.1007/s40262-016-0491-3

[8] Elens L Bouamar R Hesselink DA A new functional CYP3A4 intron 6 polymorphism significantly affects tacrolimus pharmacokinetics in kidney transplant recipients. Clin Chem. 2011;57:1574–1583. 21903774 10.1373/clinchem.2011.165613

[9] Moes DJ van der Bent SA Swen JJ Population pharmacokinetics and pharmacogenetics of once daily tacrolimus formulation in stable liver transplant recipients. Eur J Clin Pharmacol. 2016;72:163–174. 26521259 10.1007/s00228-015-1963-3 PMC4713720

[10] Haufroid V Mourad M Van Kerckhove V The effect of CYP3A5 and MDR1 (ABCB1) polymorphisms on cyclosporine and tacrolimus dose requirements and trough blood levels in stable renal transplant patients. Pharmacogenetics. 2004;14:147–154. 15167702 10.1097/00008571-200403000-00002

[11] Press RR Ploeger BA den Hartigh J Explaining variability in tacrolimus pharmacokinetics to optimize early exposure in adult kidney transplant recipients. Ther Drug Monit. 2009;31:187–197. 19258929 10.1097/FTD.0b013e31819c3d6d

[12] Degraeve AL Bindels LB Haufroid V Tacrolimus pharmacokinetics is associated with gut microbiota diversity in kidney transplant patients: results from a pilot cross-sectional study. Clin Pharmacol Ther. 2024;115:104–115. 37846607 10.1002/cpt.3077

[13] Waring RH. Cytochrome P450: genotype to phenotype. Xenobiotica. 2020;50:9–18. 31411087 10.1080/00498254.2019.1648911

[14] Kim IW Han N Burckart GJ . Epigenetic changes in gene expression for drug-metabolizing enzymes and transporters. Pharmacotherapy. 2014;34:140–150. 24166985 10.1002/phar.1362

[15] von Elm E Altman DG Egger M The Strengthening the Reporting of Observational Studies in Epidemiology (STROBE) statement: guidelines for reporting observational studies. J Clin Epidemiol. 2008;61:344–349. 18313558 10.1016/j.jclinepi.2007.11.008

[16] Pratt VM Cavallari LH Fulmer ML CYP3A4 and CYP3A5 genotyping recommendations: a joint consensus recommendation of the association for molecular pathology, clinical pharmacogenetics implementation Consortium, college of American Pathologists, Dutch pharmacogenetics working group of the royal Dutch pharmacists association, European society for pharmacogenomics and personalized therapy, and pharmacogenomics knowledgebase. J Mol Diagn. 2023;25:619–629. 37419245 10.1016/j.jmoldx.2023.06.008 PMC10565868

[17] Chavant A Fonrose X Gautier-Veyret E Variability of tacrolimus trough concentration in liver transplant patients: which role of inflammation? Pharmaceutics. 2021;13:1960. 34834375 10.3390/pharmaceutics13111960 PMC8623792

[18] Miranda TB Jones PA. DNA methylation: the nuts and bolts of repression. J Cel Physiol. 2007;213:384–390. 10.1002/jcp.21224 17708532

[19] Vyhlidal CA Bi C Ye SQ Dynamics of cytosine methylation in the proximal promoters of CYP3A4 and CYP3A7 in pediatric and prenatal livers. Drug Metab Dispos. 2016;44:1020–1026. 26772622 10.1124/dmd.115.068726

[20] Dannenberg LO Edenberg HJ. Epigenetics of gene expression in human hepatoma cells: expression profiling the response to inhibition of DNA methylation and histone deacetylation. BMC Genomics. 2006;7:181. 16854234 10.1186/1471-2164-7-181 PMC1574318

[21] Kacevska M Ivanov M Wyss A DNA methylation dynamics in the hepatic CYP3A4 gene promoter. Biochimie. 2012;94:2338–2344. 22906825 10.1016/j.biochi.2012.07.013

[22] Brussee JM Vet NJ Krekels EHJ Predicting CYP3A-mediated midazolam metabolism in critically ill neonates, infants, children and adults with inflammation and organ failure. Br J Clin Pharmacol. 2018;84:358–368. 29072785 10.1111/bcp.13459 PMC5777436

[23] Wielscher M Mandaviya PR Kuehnel B DNA methylation signature of chronic low-grade inflammation and its role in cardio-respiratory diseases. Nat Commun. 2022;13:2408. 35504910 10.1038/s41467-022-29792-6 PMC9065016

[24] Hebert MF. Contributions of hepatic and intestinal metabolism and P-glycoprotein to cyclosporine and tacrolimus oral drug delivery. Adv Drug Deliv Rev. 1997;27:201–214. 10837558 10.1016/s0169-409x(97)00043-4

