# Supplementary material for: Effect of CYP3A4 Methylation on Tacrolimus Pharmacokinetics
Source: Ther Drug Monit. 2025 Jul 8;47(6):e76–81. doi: 10.1097/FTD.0000000000001351 (PMC12588635; doi:10.1097/FTD.0000000000001351)
Supplement: Supplementary file 1 [file tdm-47-e76-s001.docx]

# Supplemental data file : Effect of *CYP3A4* methylation on tacrolimus pharmacokinetics, reported by Koudijs et al.


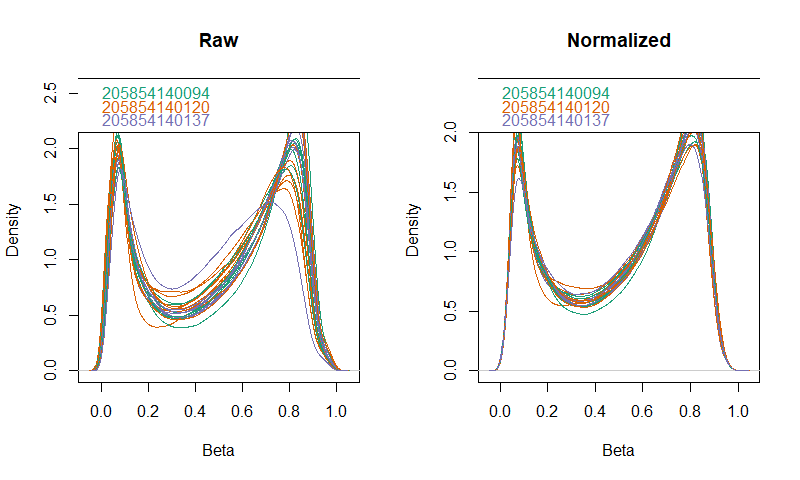


*Supplementary Fig. 1:
Distribution of Beta values before and after quantile normalization.
The different colors represent the three different slides.*


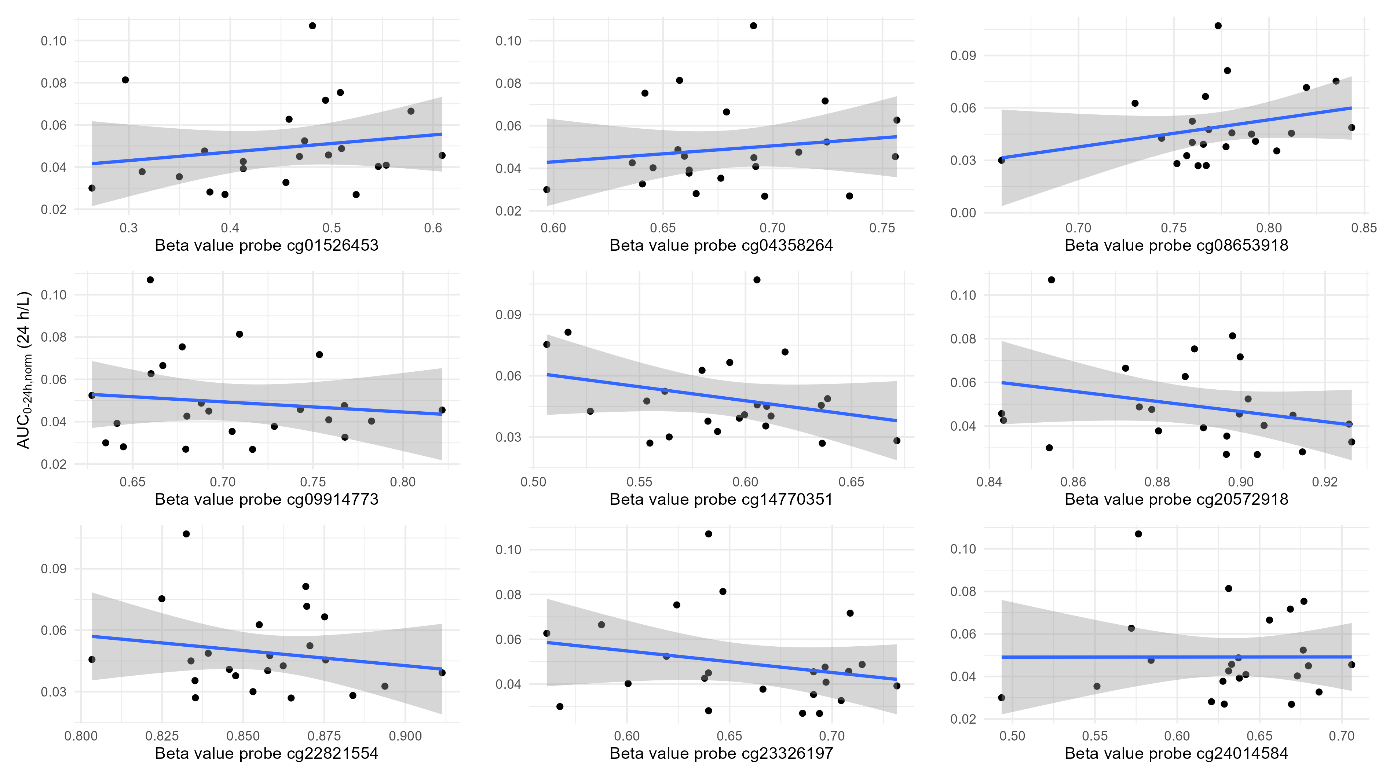


*Supplementary Fig. 2:*Area under the concentration versus time curve (AUC)_0-24h,norm_ plotted against probe methylation for the nine non-statistically significant probes. *Note: AUC_0-24h,norm_ = Dose-normalized AUC_0-24h_.*

*Supplementary Table 1:
Univariate linear model results from the investigated covariates on* the area under the concentration versus time curve *(*AUC)_0-24h,norm_*.*

| Coefficient | Estimate ± Std. error | P-value |  |
| --- | --- | --- | --- |
| Prednison/Prednisolone use | -0.007 ± 0.01 | 0.46 |  |
| Hematocrit level | 0.06 ± 0.09 | 0.52 |  |
| log(ALAT) | 0.01 ± 0.005 | 0.05 |  |
| CRP > 49 mg/L (N = 1) | -0.02 ± 0.02 | 0.26 |  |
| Recipient *CYP3A4**22 C/T genotype (N = 3) | 0.02 ± 0.01 | 0.07 |  |
| Donor *CYP3A4**22 C/T genotype (N = 4) | 0.02 ± 0.01 | 0.11 |  |

*Abbreviations: ALAT, alanine aminotransferase ;CRP, C-reactive protein*

*Supplementary Table 2:
Base pair start, end and strand of CYP3A4 methylation probes
conforming to genome assembly GRCh38 (hg38).*

| Illumina Probe ID | Base pair start | Base pair end | Strand |
| --- | --- | --- | --- |
| cg01526453 | 99783899 | 99783901 | − |
| cg04358264 | 99757580 | 99757582 | + |
| cg08653918 | 99781077 | 99781079 | + |
| cg09914773 | 99785600 | 99785602 | + |
| cg14770351 | 99778871 | 99778873 | − |
| cg19046783 | 99784462 | 99784464 | + |
| cg20572918 | 99785626 | 99785628 | + |
| cg22821554 | 99785531 | 99785533 | − |
| cg23326197 | 99784746 | 99784748 | − |
| cg24014584 | 99763262 | 99763264 | − |

*Supplementary Table 3: Inter-probe Pearson’s correlation coefficients of Beta values.*

|  | cg01526453 | cg04358264 | cg08653918 | cg09914773 | cg14770351 | cg19046783 | cg20572918 | cg22821554 | cg23326197 | cg24014584 |
| --- | --- | --- | --- | --- | --- | --- | --- | --- | --- | --- |
| cg01526453 | 1 |  |  |  |  |  |  |  |  |  |
| cg04358264 | 0.37 | 1 |  |  |  |  |  |  |  |  |
| cg08653918 | 0.47 | 0.26 | 1 |  |  |  |  |  |  |  |
| cg09914773 | 0.36 | 0.19 | 0.38 | 1 |  |  |  |  |  |  |
| cg14770351 | 0.37 | 0.20 | 0.17 | 0.19 | 1 |  |  |  |  |  |
| cg19046783 | 0.44 | −0.06 | 0.33 | −0.14 | −0.30 | 1 |  |  |  |  |
| cg20572918 | 0.19 | 0.28 | 0.25 | 0.29 | 0.28 | −0.24 | 1 |  |  |  |
| cg22821554 | −0.03 | 0.03 | −0.25 | −0.04 | 0.14 | −0.05 | 0.39 | 1 |  |  |
| cg23326197 | 0.10 | 0.13 | 0.55 | 0.44 | 0.29 | 0.03 | 0.21 | 0.07 | 1 |  |
| cg24014584 | 0.66 | 0.23 | 0.54 | 0.41 | 0.14 | 0.39 | 0.48 | 0.24 | 0.30 | 1 |
